# Genetic Variants Linked to Opioid Addiction: A Genome-Wide Association Study

**DOI:** 10.3390/ijms252312516

**Published:** 2024-11-21

**Authors:** Shailesh Kumar Panday, Vijay Shankar, Rachel Ann Lyman, Emil Alexov

**Affiliations:** 1 Department of Physics and Astronomy, Clemson University, Clemson, SC 29634, USA; spanday@clemson.edu; 2 Center for Human Genetics, Clemson University, Greenwood, SC 29646, USA; vshanka@clemson.edu (V.S.); ralyman@clemson.edu (R.A.L.)

**Keywords:** case-control genome-wide association study, protein–protein interaction network, genetic variants, functional enrichment analysis

## Abstract

Opioid use disorder (OUD) affects millions of people worldwide. While it is known that OUD originates from many factors, including social and environmental factors, the role of genetic variants in developing the disease has also been reported. This study aims to investigate the genetic variants associated with the risk of developing OUD upon exposure. Twenty-three subjects who had previously been given opioid-based painkillers to undergo minor surgical treatment were recruited at Prisma Health Upstate clinic and elsewhere. Eleven were considered nonpersistent opioid users (controls), and 12 were persistent opioid users (cases) at the time of sample collection after an initial surgery. The subjects were asked to provide saliva samples, which were subjected to DNA sequencing at Clemson University Center for Human Genetics, and variant calling was performed. The genome-wide association studies (GWASs) for genes known to be associated with OUD resulted in 13 variants (intronic or SNV) with genome-wide significance (raw *p*-value < 0.01) and two missense variants, rs6265 (p.Val66Met in BNDF isoform a) and rs1799971 (p.Asn40Asp) in *OPRM1*, previously reported in the literature. Furthermore, extending the GWASs to find all genomic variants and filtering the variants to include only variants found in cases (persistent opioid users) but not in controls (nonpersistent opioid users) resulted in 11 new variants (*p*-value < 0.005). Considering that OUD is a complex disease and the effect might come from different variants in the same genes, we performed a co-occurrence analysis of variants on the genes. We identified eight additional genes that harbor multiple variants, including four genes: *LRFN3*, *ZMIZ1*, *RYR3*, and *OR1L6*, with three or more variants in the case subjects but not in the control individuals. The performed PPI network construction, along with functional enrichment, indicated that the variants occur in calcium signaling, circadian entrainment, morphine addiction, alcoholism, and opioid signaling pathways, which are closely related to OUD or addiction in general.

## 1. Introduction

Opioid use disorder (OUD) affects millions of people worldwide, and its immense and multifaceted adverse socio-economic impacts affect individuals, families, communities, and economies [1,2]. The impacts involve drug overdose deaths [3], increased healthcare costs [4], low productivity [5], heightened criminal justice involvement [6,7], and broader societal challenges. Human studies consistently show that the heritability (a measure of how well differences in people’s genes account for differences in their traits) of opioid addiction is substantial [8,9]. Twin studies estimate the heritability of OUD to be between 40% and 60% [8,9]. Family studies also suggest that first-degree relatives of individuals with OUD have a higher risk of developing the disorder [10]. Identifying genetic variants and genes linked to elevated risks can be used to develop a better understanding of the biology of OUD. It can be further utilized in screening people for their genetic susceptibility to OUD and avoiding prescribing opioid-based painkillers to limit the risks of developing OUD. The two popular approaches to association studies are (a) candidate gene studies, as hypothesis-driven studies that analyze genes with known connections to a phenotype, and (b) GWASs, as statistical analyses of polymorphisms across a genome for association studies. Both approaches have been used to study genetic association with OUD.

Several candidate gene studies aiming to identify the genetic variants related to OUD have reported several genetic variants on genes. For example, the mu-opioid receptor 1 gene (*OPRM1*) and the variant rs1799971 (A118G) have been associated with differences in opioid effects and addiction risk [11,12,13]. Studies show that individuals with the G allele might experience altered drug effects and a higher risk of OUD [14]. Likewise, the dopamine receptor D2 gene (*DRD2*) has been implicated in the reward system’s role in addiction. Variants such as the Taq1A polymorphism are associated with an increased risk of addiction due to their impact on dopamine receptor availability and function [15,16,17,18]. The delta-opioid receptor (DOR) encoded by the *OPRD1* gene has been investigated in the context of opioid addiction, although it is less studied compared to the mu-opioid receptor [19]. Research indicates that DOR plays a role in modulating mood, anxiety, and stress responses, all of which are factors in addiction vulnerability. Variants in the *OPRD1* gene have been linked to altered responses to opioids, suggesting that DOR could influence individual susceptibility to OUD [19,20]. Brain-derived neurotrophic factor (*BDNF*) is a critical neurotrophin for brain plasticity, learning, and memory [21]. Alterations in *BDNF* expression and function have been associated with various neuropsychiatric conditions, including addiction [22]. Studies suggest that *BDNF* plays a role in the brain’s reward system and its response to opioids. Genetic variations in the *BDNF* gene might influence the risk of developing OUD by affecting neural plasticity and resilience to stress [18,23].

A few case-control genome-wide association studies (GWASs) have also been reported using varied cohorts comprising populations of varied ethnicity, sex, and opioid exposure [24,25,26,27,28]. For example, Nielsen DA et al. performed a GWAS on subjects of European origin with 104 heroin-dependent patients and 101 controls [24]. They compared the frequencies of 10,000 SNPs but failed to find any significant associations. However, they extended the cohort comprising subjects of African and European descent and a larger pool of 100,000 SNPs in 325 methadone-maintained heroin addicts and 250 controls [25]. After the multiple testing corrections, only a single intergenic variant, rs10494334, was significant in European subjects, with none in subjects of African descent.

Gelernter et al. reported a GWAS on 5432 African-American and 6877 European-American subjects [26]. Analysis using the Diagnostic and Statistical Manual of Mental Disorders 4th edition (DSM-IV) symptom counts for opioid dependence or case-control status was performed on subgroups and meta-analysis on the entire cohort. In the final meta-analysis, the variant rs62103177 in *KCNG2* was genome-wide significant. Other variants, such as rs60349741 in *KCNC1* and rs114070671 in *APBB2*, had genome-wide significance in combined analysis but only nominally in meta-analysis [26].

Defining controls merely based on the DSM definition has the caveat that the subjects not exposed to opioids cannot develop OUD, even though they carry the risk-associated variants. In a study of European Americans comprising 1290 cases and 1768 opioid-exposed controls, an SNP, rs12442183 ( 110 kb downstream to *RGMA*), was found to be significantly associated with OUD [27]. Further, microarray data analysis suggested that rs12442183 is an expression quantitative trait locus (eQTL) for the *RGMA* gene, whereby eQTL is a genomic locus that regulates the mRNA or protein expression level. However, previously reported variants on *KCNG2*, *KCNC1*, and *APBB2* did not have a significant association despite the study using the overlapping sample with the previous study [27,29].

In another study comparing daily injection opioid users (cases: n = 1,167) versus opioid abusers who never injected opioids daily (controls: n = 161), the variant rs1436175 in the gene *CNIH3* showed genome-wide significance. Five other variants—rs10799590, rs12130499, rs298733, rs1436171, and rs1369846—in *CNIH3* reached genome-wide significance in a meta-analysis of the discovery cohort and two independent populations [28,29].

When consolidated together, the aforementioned studies [24,25,26,27,28] indicate that clear external replication of GWAS findings is rare due to the following factors: (a) OUD is a complex psychiatric disease with relatively low heritability, and there is no single variant with a large effect size that can be detected in small cohorts; (b) the cohort sample size in numerous studies (e.g., Nielsen DA et al., [25] with 104 heroin-dependent patients and 101 controls) was very small for a GWAS, which typically requires large sample sizes for statistical power, in contrast to those for legal substance use disorders; (c) there was considerable phenotypic heterogeneity across samples in published work relevant to opioid use [29]; (d) OUD is likely to be highly polygenic, and individual loci contributions are too small for detection in studies with limited sample size.

With the above issues in mind, we present a case-control study of 23 individuals who had been previously given opioid-based painkillers to undergo minor surgical treatment and were recruited at Prisma Health Upstate clinic and elsewhere. Eleven were considered nonpersistent opioid users (controls), and 12 were persistent opioid users (cases) after initial surgery and at the time of the sample collection (Section 4.1). The subjects were asked to provide saliva samples, which were subjected to DNA sequencing at Clemson University Center for Human Genetics, and variant calling was performed (Section 4.2). The variants are analyzed to identify their association with OUD. The goal of this investigation is to check whether persistent opioid users’ DNA harbors mutations that are not present in nonpersistent opioid users and, thus, identify genes for which the wild-type properties are perturbed by mutations, which leads to susceptibility to OUD upon exposure.

## 2. Results

The first task of our study is to check whether previously reported variants can be found in our cohort. For this purpose, we compiled a list of genes harboring variants reported to have associations with OUD (Table 1). Subsequently, we present the results of variants found on the listed genes in our present study. Two of the variants with coding consequence (rs6265 in *BDNF* and rs1799971 in *OPRM1*) show some indication of the association to OUD, although the *p*-values of < 0.3 are nonsignificant. The search is then extended to variants with no direct coding consequences on these genes, and several such variants on *DRD3*, *KCNG2*, and *NRXN3* are found with significance (*p*-values < 0.01) for the association with OUD (Section 2.1). The next task is to search for genetic variants across the whole genome, where the alternate alleles are exclusively found in cases but not in any of the control subjects (Section 2.2). We hypothesize that several variants on the same gene might be working together to compromise the function of a gene, whereby we performed a co-occurrence analysis of variants on the genes. In co-occurrence analysis, multiple variants on the same genes are assumed to have additive impairments on the gene’s function, and these variants are grouped and analyzed for their functional consequences. This analysis identified *LRFN3*, *ZMIZ1*, *RYR3*, and *OR1L6* with three or more variants on them and an alternate allele frequency sum greater than or equal to 14 (Co-occurrence of Variants on Genes and Association with OUD Section), which might potentially have an association with OUD.

OUD is a complex phenotype that might not result from a single variant or single gene but rather a network of gene/protein interactions that are perturbed due to several variants on multiple genes. To investigate this, we also performed a network analysis of protein–protein interaction (PPI) and the genes harboring single or multiple variants suggesting an association with OUD. We identified a large, connected component of PPIs involving more than half of the genes of interest (GOI) (Section 2.3). Finally, we perform a gene function enrichment and pathway analysis to identify the molecular processes, biological function, and interaction pathways that might be affected due to the presence of variants in the cases but not in controls and discuss the results (Section 2.4).

### 2.1. Variants on Genes Reported in the Literature with Association to OUD

We begin our association analysis of OUD with the set of genes reported to be associated with OUD. Thus, we analyzed gene variants (Table 1) to test their association with OUD in our samples. We extracted the variants within extended gene loci. For each gene, an extended region with 5 kb upstream and 5 kb downstream, including the gene boundary, is defined. The effects of variants are predicted using the Variant Effect Predictor (VEP) version 111 [37] for human genome assembly GRCh38.

A total of 6,903 (including 67 novel versions) variant effects were predicted, comprising one *stop_gained*, one *inframe_insertion*, one *inframe_deletion*, 58 *missence_variant*, and 56 *synonymous_variant* with coding consequences. Further association testing of these variants was performed using PLINK v1.90b7.2 [38]. However, none of the variants with coding consequences could reach the genome-wide association significance (raw *p*-value < 0.01). By contrast, 13 variants (intronic or SNV) showed genome-wide significance, considering only a raw *p*-value of <0.01 (Table 2). However, due to the limited cohort size, upon adjusting the *p*-values for multiple corrections, all became nonsignificant with a 95% confidence interval. As the validation of intronic variants’ association with OUD requires further study, we focused on only variants with the most severe consequence (coding consequences), although none showed significance for genome-wide association with OUD. However, two variants, rs6265 (NC_000011.10:g.27658369C>T) on *BDNF*, a missense coding variant (p.Val66Met in BNDF isoform a, previously reported [18]), and rs1799971, a missense variant on *OPRM1* (c.118A > G) changing p.Asn40Asp in mu-opioid receptor (also previously reported [11,12,13]), have a raw *p*-value of <0.30, reflecting a nonsignificant trend. The large *p*-value of these variants’ association with OUD can be attributed to the small cohort used here.

The variants with no direct coding consequence that were found to be significant in the extended gene loci of the genes listed in Table 1 are shown in Table 2. These variants might have an impact on the genes by altering their expression. We found some variants, although not the listed variants, in genes *DRD3* [31,32], *KCNG2* [26,30], and *NRXN3* that have been reported to have an association with OUD [35,36].

### 2.2. Genes with Alternate Allele Exclusively in Cases but Not in Controls

We extended our search to include all genomic variants found in the samples. The variant effect is predicted using VEP version 111 [37] for 20,910 variants found in the sample. Afterward, all variants are ranked based on the severity of the predicted consequence and filtered to satisfy two requirements: (a) only variants for which all controls have homozygous reference alleles, and (b) only variants in the OUD group that have the alternative alleles. A variant’s association with phenotype OUD is considered significant when the raw *p*-value is less than 0.05. It resulted in 158 variants, with significance within 126 genes. However, due to the limited cohort size, upon adjusting the *p*-values for multiple corrections, all became nonsignificant with a 95% confidence interval. In the case of these variants with significance, the alternate allele frequency ranged from 11 to four in the case samples and zero in the controls. A list of such variants with an association with OUD with only significance (raw *p*-value < 0.005) is provided in Table 3.

#### Co-Occurrence of Variants on Genes and Association with OUD

Thus far, our focus has been placed on finding common genetic variants that might be associated with OUD. However, OUD is a complex phenotype that might result from the altered function of one or more proteins, which could be caused by different variants within the same proteins, not necessarily identical variants in the affected individuals. It is also possible that there are multiple mechanisms and disruptions of pathways that can result in OUD. This led us to focus on proteins or genes and their combinations instead of individual variants. Considering this, we analyzed the co-occurrence of multiple variants within the same gene/protein. Since the 158 identified variants associated with OUD are present in 126 genes, 18 of these genes have multiple variants on them. For each of these 18 genes that harbor multiple variants, the alternate allele count of all gene variants exclusively in cases is aggregated by summing, considering significance (raw *p*-value < 0.05). Note that none of the variants on a given gene has any alternate allele in the controls. Among the 18 genes with multiple variants exclusively in cases, 12 include genes with two variants, three genes with three variants, and three genes with more than three variants.

The following genes harbor the most severe consequence (transcript ablation, splice acceptor variant, splice donor variant, stop gained/lost, frameshift variant, start lost, transcript amplification, feature elongation, or feature truncation) variants, with an alternate allele frequency sum greater than or equal to 10: *ZMIZ1*, *LRFN3*, *OR1L6*, *RYR3*, *PWWP2B*, *ZNF92*, *CYP4F12*, and *NUTM2D*. The number of variants, the counts of alternate alleles in cases, and the list of predicted consequences for the variants are provided in Table 4. Considering the significant raw *p*-values of the genes listed in Table 3 or Table 4, we see a total of 19 genes, and we will call these genes of interest (GOI) in further analysis.

Thus far, none of the genes/proteins listed in Table 4 have been directly associated with addiction. However, some of these have been known to be associated with functions relevant to OUD. For example, LRFN3 (Leucine Rich Repeat And Fibronectin Type III Domain Containing 3), also known as SALM4 (Synaptic Adhesion-Like Molecule 4), is part of the LRFN family, which is involved in synaptic adhesion and regulation of excitatory synapses [39]. SALMs, including LRFN3/SALM4, play important roles in synaptic development and plasticity [40] as critical processes in learning, memory, and behavior, all of which are relevant to addiction. Similarly, RYR3 (Ryanodine Receptor 3) is part of the ryanodine receptor family, which functions as an intracellular calcium channel that releases calcium from the endoplasmic reticulum into the cytoplasm [41,42]. Calcium signaling is critical for various cellular processes, including neuronal activity, synaptic plasticity, and neurotransmitter release [43]. ZMIZ1 (Zinc Finger MIZ-Type Containing 1) is a transcriptional coactivator that interacts with the androgen receptor and other transcription factors [44]. The androgen receptor has been implicated in reward-related behaviors, including aggression and stress responses, which can influence addiction [45]. In summary, the genes listed in Table 4 can potentially influence addictive behaviors directly or indirectly and in individual- or cohort-specific manners. This led us to further analyze the interaction networks of these genes and investigate their collective behaviors and plausible linkage to opioid addiction.

### 2.3. PPI Network Analysis of Genes of Interest

**Figure 1:**
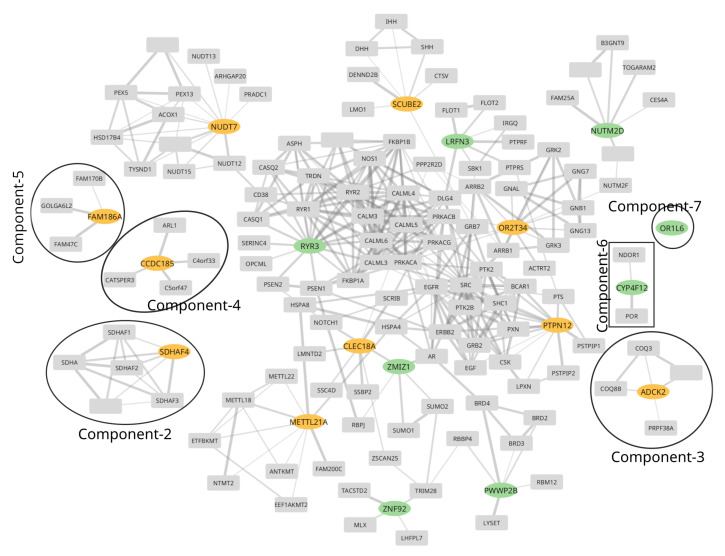
Protein–protein interaction (PPI) network of high-confidence (normalized interaction score > 0.8, details in Section 4.4) PPIs subset from the STRING database. The genes harboring a single variant with a very high alternate allele count exclusively (Table 3) in cases are shown with orange oval nodes, while genes harboring multiple variants with a high alternate allele count exclusively in cases (Table 4) are shown in green oval nodes/genes. All other gray nodes are either immediate neighbors (directly interacted by PPIs) of green or orange nodes or nodes having at least two neighbors in the list of selected nodes/genes. Finally, the subnetwork is created using selected nodes and all edges.

To gain insight into the functions involving the proteins in GOI, we built a PPI network based on the STRING database [46], as detailed in Section 4.4. We observe a total of seven connected components. Connected components 2 to 7 are small and are shown in Figure 1, while all other genes form a large connected component, marked as component 1. Component 1 comprises six genes (*SCUBE2*, *PTPN12*, *NUDT7*, *METTL21A*, *CLEC18A*, and *OR2T34*), listed in Table 3 and five genes (*ZMIZ1*, *LRFN3*, *RYR3*, *PWWP2B*, and *ZNF92*) harboring multiple variants listed in Table 4. Note that the genes listed in these two tables are mutually exclusive. This indicates that genes in component 1 are functionally interdependent, and any perturbation to this network of PPIs can potentially be associated with altered function and, thereby, some phenotype. To further investigate this hypothesis of perturbation of the PPI network and association with OUD, we performed a functional enrichment analysis of the genes in the network.

### 2.4. Gene Ontology and Functional Enrichment Analysis

**Figure 2:**
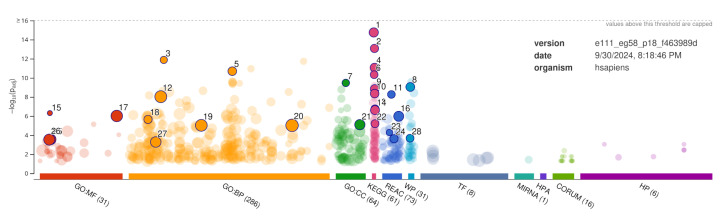
Summary of gene ontology and functional enrichment of genes in PPIs network of GOIs performed using the g:Profile web server. The highly significant (considering the adjusted *p*-value) but nonredundant terms in the enrichment are annotated. The description of the annotation labels regarding terms is provided in Table 5.

The functional enrichment analysis was performed using the g:Profiler web service [47], and its results are summarized in Figure 2 and Table 5.

From the enrichment analysis, the calcium transport, regulation and binding, enzyme binding, transmembrane transporter binding, and signaling receptor binding emerge as the high-confidence molecular function from gene ontology. Signaling and amino-acid modification are the main biological functions. The GO:cellular component is the membrane and channels, which host most of the signaling and transmembrane proteins to carry the signaling in cells.

The KEGG pathway analysis shows enrichment in calcium signaling pathways, circadian entrainment, dopaminergic synapses, morphine addiction, and alcoholism (Figure 2 and Table 5). This further supports the notion that the variants of GOIs are impairing or abolishing the function of the proteins, which, in turn, compromises the protein’s normal function in the PPI network since these proteins are involved in crucial reward pathways and addiction. Therefore, the association of these genetic variants with OUD risk is highly plausible, and further study is needed to confirm this.

## 3. Discussion

OUD is a complex phenotype with significant heritability estimated at between 40% and 60%. The heritability raises the genetic susceptibility of individuals carrying the associated variants in their genome. In the past, several candidate gene studies and GWASs have implicated the association of variants on genes with OUD, although they have rarely been replicated in other studies. It has been highlighted that this situation can be due to the following limitations of the studies: (a) the cohort sizes used are relatively small compared to those used for substance use addiction; (b) the DSM definitions have been used to label controls, which might not be appropriate and, thus, dilute the association results; (c) the ethnicity of populations of cohorts varied in different studies, which brings another variability in the study. Another limitation of some previous studies is that the controls were not exposed to substances of interest. Such controls could potentially carry genetic variants, making them predisposed to OUD, although they have never been exposed to drugs.

To address the above-mentioned deficiency, we performed a genome-wide association study of a cohort of 23 samples, including 12 cases and 11 controls, where all controls were given opioid-based painkillers. In our study, we could find a weak association for two variants, one on *BDNF* and the other on *OPRM1*, when our focus was on variants only with coding consequences. Upon extending the search to variants with no direct coding consequence, we identified variants of *DRD3*, *KCNG2*, and *NRXN3* genes with significant association with OUD. Interestingly, these genes have been predicted to have an association with OUD. This indicates that these variants might be altering the expression of the protein and thereby indirectly affecting the OUD phenotype.

Next, we expanded our search space for the variants to the entire genome, although a genome-wide search yields many variants. Thus, only variants with coding consequences are prioritized in the association analysis. We chose only variants with alternate alleles in case subjects and predicted them to have an association with OUD with significance (Table 3). Thus far, none of the genes listed in Table 3 have been directly linked to addiction. However, in a study of attention-deficit hyperactive patients, gene *MTUS2* showed the highest variation frequency [48]. Studies have suggested the association of *MTUS2* with psychological disorders [48], which might indirectly relate to addiction-like traits. However, further exploration in this regard is necessary to test this possibility.

Several variants of the same gene protein might have a cumulative effect on the gene’s function, thus altering its biological function. We used co-occurrence analysis to test this hypothesis. Our co-occurrence analysis identified *LRFN3*, *RYR3*, *ZMIZ1*, and *OR1L6* as genes harboring multiple variants exclusively in cases. From the genes with a predicted association with OUD with significance (genes of interest/GOI), a network of protein–protein interaction is extracted based on PPIs listed in the STRING database for humans. The network is further expanded by including genes directly interacting with two or more genes in the GOI list. Subsequently, the pathway and functional enrichment of the genes in the network are undertaken to find the pathways with significant enrichment for the genes and their association with the OUD phenotype. We observe considerable enrichment for terms such as calcium signaling pathway, circadian entrainment, dopaminergic synapse, oxytocin signaling pathway, estrogen signaling pathway, morphine addiction, alcoholism, and opioid signaling, which are closely related to OUD or addiction in general.

The small sample size available for the study is one of the limitations based on which none of the variants could reach genome-wide significance upon invoking multiple hypothesis corrections and adjusting the raw *p*-values. The small sample size also limits ascertaining association differences due to variations in age (a wide range of 36 to 84 years, mean: 66 years), sex, and ethnicity in the sample. The results of the study should be interpreted considering this limitation, and further validation of our results would require a larger sample size.

## 4. Materials and Methods

### 4.1. Sample Collection

Local Institutional Review Board approval was obtained prior to subjects’ recruitment and enrollment, and written informed consent was obtained from all eligible participants. Eligible subjects were those over 18 who had previously undergone treatment for minor surgery and provided informed consent. Subjects were excluded if they had an active oral lesion, a history of opioid abuse before the treatment of their injury, or a history of opioid use within 1 year of treatment for their injury. Subjects who provided informed consent were subsequently asked to provide a saliva sample for DNA analysis. Twenty subjects were recruited and enrolled between November 2022 and June 2023, and their saliva was collected during a visit to a medical facility. Twelve subjects were persistent opioid users at the time of enrollment and saliva sample collection, whereas there were only eight nonpersistent opioid users. To make the set balanced, we collected additional samples from researchers from the corresponding author’s (EA) department, who had previously been sedated for minor surgery but were not persistent users. Thus, the number of cases of nonpersistent users is 11, similar to that of persistent users. Persistent opioid users are individuals requesting opioid prescriptions for more than 6 months after the surgery.

Age, gender, and race demographics were recorded at the enrollment visit, in addition to details of their orthopedic injuries. The average age of all enrolled participants was 66 years, with a minimum age of 36 and a maximum age of 84. There were eight females and three males in the nonpersistent opioid user group and seven females and five males in the persistent opioid user group. All participants are Caucasians.

### 4.2. DNA Sequencing and Genotyping

The samples’ DNA was extracted using a modified protocol with Quick-DNA Microprep Plus Kit, Zymo Research Corporation, Irvine, CA, USA. The libraries were constructed using 500 ng of extracted DNA with the Revelo DNA-Seq Enz for MagicPrep Kit, Tecan Genomics Inc., Redwood City, CA, USA, on the MagicPrep NGS Instrument, Tecan Genomics Inc., Redwood City, CA, USA. The libraries were quantified for concentration with the 1X dsDNA HS (high sensitivity) Assay Kit, Invitrogen Corporation, Carlsbad, CA, USA on the Qubit 4 Fluorometer, Invitrogen Corporation, Carlsbad, CA, USA. Library size was quantified using High Sensitivity DNA ScreenTape Analysis, Agilent Technologies, Santa Clara, CA, USA on the 4150 TapeStation System, Agilent Technologies, Santa Clara, CA, USA. The libraries were normalized to 3 nm, pooled, and sequenced on an NovaSeq 6000 System, Illumina Inc., San Diego, CA, USA using NovaSeq 6000 S4 Reagent Kit v1.5 (300 cycles), Illumina Inc., San Diego, CA, USA flow cell sequencing chemistry at Clemson University Center for Human Genetics.

Raw reads were filtered for low-quality and short reads, then aligned to human reference genome version GRCh38 to produce alignment files using the GPU-accelerated *fq2bam* module in the NVIDIA Clara Parabricks suite. Base quality recalibration was performed using the GPU-accelerated Genome Analysis Tool Kit [49] BQSR module from Parabricks suite and known variant information from the Mills and 1000 Genomes Gold Standard Indel dataset and dbSNP v138 dataset. Variant calling was performed on the recalibrated alignment files using GPU-accelerated GATK’s haplotypecaller module from the Parabricks suite and GRCh38 reference genome. Individual sample GVCFs were combined, indexed, and joint-called using the Short Variant Discovery workflow from GATK’s Best Practices [50]. Joint-called variants were hard-filtered using gold standard default values recommended by the Broad Institute [51]. Subsequently, the effects for variants are predicted using VEP version 111 [37] compared to standard human genome assembly GRCh38.

### 4.3. Variants Filtering, Effect Prediction, and Association Analysis

A subset of all variants found after variant calling was extracted into a .vcf file using the bcftools module in SAMtools [52] to analyze the association of a subset of genes listed in Table 1. The subset .vcf file is indexed and compressed using bcftools [52]. The variant effects are predicted using Ensembl Variant Effect (VEP) version 111 [37] with reference to human genome assembly GRCh38. We used PLINK v1.90b7.2 [38] to test the significance of the genotype–phenotype association, with parameter values of a 95% confidence interval, missingness per SNP at 0.1, minor-allele frequency at 0.05, a Hardy-Weinberg threshold of 0.0000001, non-founders, allow-no-sex, and keep-allele-order options.

### 4.4. PPI Network Construction and Analysis

The PPI data for humans was downloaded from the STRING database [46]. Considering the large number of human PPIs listed in STRING and varying degrees of confidence in PPIs, we started filtering only high-confidence PPIs based on their absolute *combined_score*, including only interactions with a *combined_score* greater than a defined threshold.

**Figure 3:**
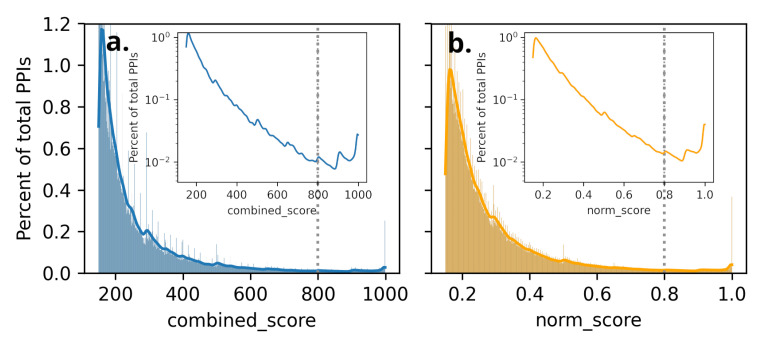
Distribution of percent of total PPIs as a function of scores: (**a**) *combined_score* and (**b**) normalized score(*norm_score*) listed in STRING database for humans. The kernel density of the percent of PPIs is shown in a logarithmic scale to highlight distributions in low-percent regions (in inset) within respective plots. The thresholds in this work are marked with dashed vertical lines in both cases.

However, we noticed that filtering based on absolute *combined_score* excluded all interactions of proteins with no partner with a *combined_score* greater than the chosen threshold. However, such filtering excluded some of the GOIs altogether. To avoid such stringent absolute *combined_score*-based filtering, we normalized the *combined_score* using the max *combined_score* for the gene among all its interacting genes. Such normalization is carried out to ensure that high-confidence PPIs can be included in a subnetwork featuring only high-confidence interactions, as per Figure 3. The percentage of PPIs keeps decreasing as a function of *combined_score* reaches a minimum of around 800. After this, it starts increasing, implying that there are high-confidence PPI pairs with scores greater than 800. Based on this, we choose *combined_score* = 800 as the threshold for unnormalized scores (Figure 3). We also observe a similar trend for normalized scores, and at around *norm_score* = 0.8, the percentage of PPI starts increasing again. Thus, we choose 0.8 as a threshold for the *norm_score* to find the filtered subset of the STRING database comprising 19,622 nodes and 204,572 edges (after removing self-loop and duplicate edges) for further analysis using Cytoscape-v3.10.2 [53].

We start with creating a subnetwork of PPIs for GOIs. Initially, the genes listed in Table 3 or Table 4 and any immediate neighbor to these are also selected. Further, any node within a distance of one and at least two neighbors in the selected nodes set is also considered part of the subnetwork to extend the network of PPIs of GOIs. Finally, the subnetwork is created using selected nodes and all edges. The created subnetwork comprises 146 nodes and 390 edges, as shown in Figure 1.

### 4.5. Gene Ontology and Functional Enrichment

The g:Profiler [47] carries the enrichment of the genes/proteins list provided as input against the data sources: gene ontology (molecular function, biological process, and cellular component), biological pathways (KEGG, Reactome, and WikiPathways), regulatory motifs in DNA (TRANSFAC and miRTarBase), protein databases (Human Protein Atlas and CORUM), and human phenotype ontology (HP). We used the list of genes present in the connected component 1 of the network for the enrichment analysis. The gene IDs are Gn148400, Gn132535, Gn139549, Gn198838, Gn157322, Gn145687, Gn089250, Gn105426, Gn103254, Gn140368, Gn172824, Gn109971, Gn166444, Gn110031, Gn129007, Gn133835, Gn137486, Gn203740, Gn188612, Gn141480, Gn141738, Gn160691, Gn237172, Gn175470, Gn050820, Gn139197, Gn153485, Gn167378, Gn152229, Gn221886, Gn206069, Gn088832, Gn120899, Gn213965, Gn204256, Gn132589, Gn197037, Gn177885, Gn116030, Gn221874, Gn176533, Gn067365, Gn214562, Gn119782, Gn178372, Gn189350, Gn198363, Gn078369, Gn169717, Gn165059, Gn137312, Gn169083, Gn244462, Gn197122, Gn162521, Gn184292, Gn142875, Gn203791, Gn143318, Gn143801, Gn198626, Gn142949, Gn196218, Gn139160, Gn166321, Gn180900, Gn168214, Gn188322, Gn188100, Gn169398, Gn166407, Gn108175, Gn141404, Gn186439, Gn146757, Gn185522, Gn183310, Gn183715, Gn080815, Gn184716, Gn100077, Gn178363, Gn173020, Gn175356, Gn072062, Gn171806, Gn171813, Gn169925, Gn169885, Gn170606, Gn164690, Gn163501, Gn162928, Gn161533, Gn160014, Gn156521, Gn150787, Gn146700, Gn146648, Gn144401, Gn141736, Gn140876, Gn089159, Gn138798, Gn141867, Gn118729, Gn137727, Gn136943, Gn136159, Gn135617, Gn130950, Gn130726, Gn127947, Gn127588, Gn108788, Gn126243, Gn112874, Gn004468, and Gn103653, where the prefix Gn is used in the listing here in place of *ENSG00000* for brevity. The enrichment query parameters are as follows: *version* e111_eg58_p18_f463989d; *date* 30 September 2024, 8:18:46 PM; *organism* hsapiens; *query length* 122; *all results* false; *ordered* false; *no iea* false; *sources* GO:MF, GO:CC, GO:BP, KEGG, REAC, TF, MIRNA, HPA, CORUM, HP, WP; *multiquery* false; *numeric ns* ENTREZGENE_ACC; *domain scope* annotated; *measure underrepresentation* false; *significance threshold method* g_SCS; *user threshold* 0.05; *no evidences* false; *highlight results* true. The results are summarized and discussed in the results section.

## 5. Conclusions

OUD is a complex disorder that has a genetic factor in addition to social and environmental factors. We investigated the genetic associations of OUD on a sample of 23 subjects who had previously taken opioid-based painkillers to undergo minor surgery. Among them, 12 subjects were considered persistent opioid users (cases), and the remaining 11 were considered the controls. While the study identified two missense variants, rs6265 (p.Val66Met in BNDF isoform a) and rs1799971 (p.Asn40Asp in OPRM1), that were previously reported, the contributions of this work to the field of OUD comprise three important considerations and findings. First, we emphasize that both the cases and the controls must be exposed to painkillers to probe their susceptibility to OUD. Indeed, an individual can have a genetic predisposition to OUD, but if never exposed to drugs, this will not be detected. Another important finding and consideration stems from the understanding that OUD is a complex disease, and the genetic component does not have to have the same signature in all affected individuals. Thus, instead of seeking identical variants in the cases, we identified variants within the same genes in the cases but not in the controls. Thirteen intronic variants in extended genic regions (−5 kb–gene-region–+5 kb) showed a raw *p*-value of <0.01 with OUD association (none reached significance upon multiple hypothesis correction due to the small sample size). Genome-wide association with OUD was performed, whereby variants with direct coding consequences were prioritized, and variants with alternate alleles only in case subjects (not controls) were filtered to result in 11 variants in 11 genes: *FAM186A*, *SCUBE2*, *CCDC185*, *MTUS2*, *PTPN12*, *ADCK2*, *NUDT7*, *METTL21A*, *SDHAF4*, *CLEC18A*, and *OR2T34*, with a raw *p*-value of < 0.005 (no multiple hypothesis correction and *p*-value adjustment). More importantly, in the co-occurrence analysis, eight genes (*ZMIZ1*, *LRFN3*, *OR1L6*, *RYR3*, *PWWP2B*, *ZNF92*, *CYP4F12*, and *NUTM2D*) harbored two or more variants with an alternate allele frequency sum of 10 or more. With these 19 genes, a network of high-confidence PPI is constructed. The network’s largest connected component contained six genes (*SCUBE2*, *PTPN12*, *NUDT7*, *METTL21A*, CLEC18A, and OR2T34) from single variants and five genes (ZMIZ1, LRFN3, *RYR3*, *PWWP2B*, and *ZNF92*) from the multivariant genes list. The genes from the network’s largest connected component are used for functional enrichment analysis, and several pathways such as calcium signaling pathway, circadian entrainment, dopaminergic synapse, morphine addiction, alcoholism, amphetamine addiction, and opioid signaling related to addiction or similar traits are enriched, suggesting the potential role of these variants on these genes in OUD. These results underscore that OUD, being a complex phenotype, may result from variations in several genes, which may not necessarily be directly related to opioid signaling pathways. Further, studies to validate the association of these genes with OUD, considering one gene at a time, should shed light on the affected pathways and molecular processes involved. However, due to the small sample size, considering multiple hypothesis-corrected *p*-values, none of the variants reached significance. Additionally, variations in age, sex, and ethnicity in the sample limit the generalizability of the association results. The results of the study should be interpreted considering the mentioned limitation. Further study on a larger sample is necessary to validate the current results.

## Figures and Tables

**Table 1:** List of genes reported in the literature harboring variants associated with OUD risk.

Gene	${\mathrm{Chr}}^{\;a}$	${\mathrm{Gene BP(beg)}}^{\;b}$	${\mathrm{Gene BP(beg)}}^{\;c}$	Variant (rsID)	Sample Population	Opioid-Exposed Controls	Reference
OPRM1	chr6	154039240	154132356	rs1799971	${\mathrm{EuA}}^{\;d}$, AfA ^*e*^	Yes	[11,12,13]
OPRD1	chr1	28812170	28871267	rs2236861	EuA, AfA; EuA, AfA	Yes; No	[12,19]
DRD2	chr11	113409605	113475398	rs1799978	${\mathrm{Eu}}^{\;f}$, ${\mathrm{NaAm}}^{\;g}$, ${\mathrm{Asn}}^{\;h}$, ${\mathrm{Per}}^{\;i}$	Yes	[18]
BDNF	chr11	27654893	27700455	rs6265	${\mathrm{Eu}}^{\;f}$, ${\mathrm{NaAm}}^{\;g}$, ${\mathrm{Asn}}^{\;h}$, ${\mathrm{Per}}^{\;i}$	Yes	[18]
APBB2	chr4	40810027	41214542	rs114070671	${\mathrm{EuAm}}^{\;j}$, ${\mathrm{AfAm}}^{\;k}$	No	[26]
KCNG2	chr18	79797938	79900100	rs62103177	EuAm, AfAm	No	[26,30]
KCNC1	chr11	17734781	17783057	rs60349741	EuAm, AfAm	No	[26]
CNIH3	chr1	224616317	224740554	rs1436175, rs10799590, rs12130499, rs298733, rs1436171, and rs1369846	${\mathrm{EuAu}}^{\;l}$	Yes	[28]
RGMA	chr15	93035271	93089211	rs12442183	EuAm	Yes	[27]
DRD3	chr3	114127580	114179052	rs324029 and rs2654754	EuAm, AfAm	No	[31,32]
DRD4	chr11	637269	640706	rs1800955	Chinese males	No	[33,34]
NRXN3	chr14	78170373	79868291	${\mathrm{rs8019381}}^{\;m}$	Caucasians	No	[35,36]

^*a*^ Chromosome; ^*b*^ Gene’s beginning base position; ^*c*^ Gene’s ending base position; ^*d*^ European ancestry; ^*e*^ African ancestry; ^*f*^ European; ^*g*^ Native South/North American; ^*h*^ Asian; ^*i*^ Persian; ^*j*^ European-American; ^*k*^ African-American; ^*l*^ European-Australian; ^*m*^ Associated to substance use disorder; Gene symbols: OPRM1—opioid receptor mu 1; OPRD1—opioid receptor delta 1; DRD2—dopamine receptor D2; BDNF—brain-derived neurotrophic factor; APBB2—amyloid beta precursor protein binding family B member 2; KCNG2—potassium voltage-gated channel modifier subfamily G member 2; KCNC1—potassium voltage-gated channel subfamily C member 1; CNIH3—cornichon family AMPA receptor auxiliary protein 3; RGMA—repulsive guidance molecule BMP co-receptor a; DRD3—dopamine receptor D3; DRD4—dopamine receptor D4; NRXN3—neurexin 3.

**Table 2:** The noncoding consequence variants reaching significance (considering a raw *p*-value < 0.01 only) with an association with OUD in the extended genes loci are listed in Table 1, as noncoding variations might influence gene expression.

${\mathrm{Chr}}^{\;a}$	${\mathrm{Variant}}^{\;b}$ or rsID	${\mathrm{BP}}^{\;c}$	Gene	${\mathrm{F\_A}}^{\;d}$	${\mathrm{F\_U}}^{\;e}$	${\mathrm{Conseqn.}}^{\;f}$	$\chi^{2}$	*p*-Value	${\mathrm{OR}}^{\;g}$
3	${\mathrm{TGAAA > T}}^{\;h}$	114142739	DRD3	0.0909	0.5455	${\mathrm{iv}}^{\;i}$	10.48	0.0012	0.0833
18	rs76838079	79873271	KCNG2	0.3182	0	iv	8.324	0.0039	NA
3	rs73232565	114124222	-	0	0.2727	-	7.527	0.0061	0
11	rs3051820	17785864	-	0.2727	0.6818	-	7.379	0.0066	0.175
4	rs1011069	41217734	-	0.2727	0	-	6.947	0.0084	NA
14	rs143010574	79227804	NRXN3	0.2727	0	${\mathrm{gutv}}^{\;j}$, iv	6.947	0.0084	NA
4	rs7695309	41216892	-	0.3636	0.0455	-	6.844	0.0089	12
14	rs7145683	79241818	NRXN3	0.3636	0.0455	gutv, iv	6.844	0.0089	12
14	${\mathrm{G > GA}}^{k}$	79242068	NRXN3	0.3636	0.0455	gutv, iv	6.844	0.0089	12
14	rs12889183	79360638	NRXN3	0.5	0.1364	iv	6.705	0.0096	6.333
14	rs11625994	79364803	NRXN3	0.5	0.1364	iv	6.705	0.0096	6.333
14	rs8008332	79365491	NRXN3	0.5	0.1364	iv	6.705	0.0096	6.333
14	rs2167150	79367835	NRXN3	0.5	0.1364	iv	6.705	0.0096	6.333

^*a*^ Chromosome; ^*b*^ rsID: if found, it is listed; otherwise, the variant is listed in this column in the form of X > Y, where X is the reference allele, and Y represents the alternate allele; ^*c*^ Base position; ^*d*^ minor-allele frequency in cases; ^*e*^ minor-allele frequency in controls; ^*f*^ variants’ consequence; ^*g*^ odds ratio; ^*h*^ copy number variation, deletion; ^*i*^ intronic variant; ^*j*^ genic upstream transcript variant; ^*k*^ insertion; Gene symbols: DRD3—dopamine receptor D3; KCNG2—potassium voltage-gated channel modifier subfamily G member 2; NRXN3—neurexin 3.

**Table 3:** The variants across the genome are ranked by consequence and filtered as per significance associated with OUD.

${\mathrm{Chr}}^{\;a}$	${\mathrm{Variant}}^{\;b}$ or rsID	${\mathrm{BP}}^{\;c}$	Gene	${\mathrm{C\_A}}^{\;d}$	Conseqn. ^*e*^	$\chi^{2}$	*p*-Value
12	rs773026868	50352078	FAM186A	11	${\mathrm{fsv}}^{\;f}$	13.25	0.000272
11	rs60494098	9091455	SCUBE2	10	${\mathrm{mv}}^{\;g}$	12.94	0.000321
1	rs10907376	223394461	CCDC185	9	mv	11.31	0.000769
13	C > A	29324631	MTUS2	8	mv	9.778	0.001766
7	rs3750050	77627396	PTPN12	7	mv	8.324	0.003912
7	rs1046515	140694787	ADCK2	7	mv	8.324	0.003912
16	rs308925	77735937	NUDT7	7	mv	8.324	0.003912
2	A > G	207613128	METTL21A	7	mv	8.324	0.003912
6	rs1048886	70579486	SDHAF4	7	mv	8.324	0.003912
16	rs3869427	69954416	CLEC18A	7	mv	8.324	0.003912
1	rs147489167	248574363	OR2T34	7	mv	8.324	0.003912

^*a*^ Chromosome; ^*b*^ rsID: if found, it is listed; otherwise, the variant is listed in this column in the form of X > Y, where X is the reference allele, and Y represents the alternate allele; ^*c*^ Base position; ^*d*^ alternate allele count in cases; ^*e*^ variants’ consequence; ^*f*^ frameshift variant; ^*g*^ missense variant; Gene symbols: FAM186A—family with sequence similarity 186 member A; SCUBE2—signal peptide, CUB domain and EGF like domain containing 2; CCDC185—coiled-coil domain containing 185; MTUS2 – microtubule-associated scaffold protein 2; PTPN12—protein tyrosine phosphatase non-receptor type 12; ADCK2—aarF domain containing kinase 2; NUDT7—nudix hydrolase 7; METTL21A—methyltransferase 21A, HSPA lysine; SDHAF4—succinate dehydrogenase complex assembly factor 4; CLEC18A—C-type lectin domain family 18 member A; OR2T34—olfactory receptor family 2 subfamily T member 34.

**Table 4:** Details of co-occurring variants on genes, where the sum of alternate allele count in cases is greater than or equal to 10 but zero in controls.

${\mathrm{Chr}}^{\;a}$	Gene	${\mathrm{\#Variants}}^{\;b}$	${\mathrm{List of C\_A}}^{\;c}$	${\mathrm{List of Consequences}}^{\;d}$
10	ZMIZ1	7	5, 5, 5, 4, 4, 4, 4	fsv ^*e*^, ${\mathrm{mv}\;}^{f}$, fsv, fsv, fsv, fsv, fsv
19	LRFN3	6	5, 5, 5,5, 5, 5	${\mathrm{id}}^{\;g}$, ${\mathrm{pav}}^{\;h}$, ${\mathrm{ii}}^{\;i}$, fsv, fsv, fsv
9	OR1L6	4	4, 4, 4, 4	mv, mv, mv, mv
15	RYR3	3	5, 5, 4	fsv, fsv, ${\mathrm{sg}}^{\;j}$ & fsv
10	PWWP2B	3	4, 4, 4	sg, mv, fsv
7	ZNF92	2	6, 6	mv, mv
19	CYP4F12	3	4, 4, 4	mv, mv, ${\mathrm{sdv}}^{\;k}$ & ${\mathrm{ntv}}^{\;l}$
10	NUTM2D	2	5, 5	mv, mv

^*a*^ Chromosome; ^*b*^ number of variants; ^*c*^ alternate allele counts in cases for each variant; ^*d*^ variants’ consequence; ^*e*^ frameshift variant; ^*f*^ missense variant; ^*g*^ inframe deletion; ^*h*^ protein-altering variant; ^*i*^ inframe insertion; ^*j*^ stop gained; ^*k*^ splice donor variant; ^*l*^ nmd transcript variant; Gene symbols: ZMIZ1—zinc finger MIZ-type containing 1; LRFN3 – leucine-rich repeat and fibronectin type III domain containing 3; OR1L6—olfactory receptor family 1 subfamily L member 6; RYR3—ryanodine receptor 3; PWWP2B—PWWP domain containing 2B; ZNF92—zinc finger protein 92; CYP4F12—cytochrome P450 family 4 subfamily F member 12; NUTM2D—NUT family member 2D.

**Table 5:** Description of annotated terms shown in Figure 2. Terms relevant to addiction are shown in underlined text.

ID	Source	Term ID	Term Name	$P_{\mathrm{adj}}$ (Query)
1	KEGG	KEGG:04020	Calcium signaling pathway	$1.780\times 10^{-15}$
2	KEGG	KEGG:04713	Circadian entrainment	$8.371\times 10^{-14}$
3	GO:BP	GO:0014808	Release of sequestered calcium ion into cytosol via sarcoplasmic reticulum	$1.334\times 10^{-12}$
4	KEGG	KEGG:04728	Dopaminergic synapse	$8.377\times 10^{-12}$
5	GO:BP	GO:0051208	Sequestering of calcium ion	$2.017\times 10^{-11}$
6	KEGG	KEGG:04340	Hedgehog signaling pathway	$4.704\times 10^{-11}$
7	GO:CC	GO:0033017	Sarcoplasmic reticulum membrane	$3.405\times 10^{-10}$
8	WP	WP:WP3929	Chemokine signaling pathway	$9.043\times 10^{-10}$
9	KEGG	KEGG:04921	Oxytocin signaling pathway	$1.489\times 10^{-9}$
10	KEGG	KEGG:04915	Estrogen signaling pathway	$4.481\times 10^{-9}$
11	REAC	REAC:R-HSA-5578775	Ion homeostasis	$5.472\times 10^{-9}$
12	GO:BP	GO:0010646	Regulation of cell communication	$9.459\times 10^{-9}$
13	KEGG	KEGG:05032	Morphine addiction	$1.816\times 10^{-7}$
14	KEGG	KEGG:05034	Alcoholism	$2.403\times 10^{-7}$
15	GO:MF	GO:0005219	Ryanodine-sensitive calcium-release channel activity	$4.909\times 10^{-7}$
16	REAC	REAC:R-HSA-9006934	Signaling by receptor tyrosine kinases	$1.062\times 10^{-6}$
17	GO:MF	GO:0140096	Catalytic activity, acting on a protein	$9.842\times 10^{-7}$
18	GO:BP	GO:0006942	Regulation of striated muscle contraction	$2.257\times 10^{-6}$
19	GO:BP	GO:0036211	Protein modification process	$9.419\times 10^{-6}$
20	GO:BP	GO:1901564	Organonitrogen compound metabolic process	$9.464\times 10^{-6}$
21	GO:CC	GO:0098797	Plasma membrane protein complex	$8.009\times 10^{-6}$
22	KEGG	KEGG:05031	Amphetamine addiction	$6.351\times 10^{-6}$
23	REAC	REAC:R-HSA-180292	GAB1 signalosome	$5.328\times 10^{-5}$
24	REAC	REAC:R-HSA-111885	Opioid signaling	$2.349\times 10^{-4}$
25	GO:MF	GO:0005509	Calcium ion binding	$2.788\times 10^{-4}$
26	GO:MF	GO:0005102	Signaling receptor binding	$2.920\times 10^{-4}$
27	GO:BP	GO:0009725	Response to hormone	$5.256\times 10^{-4}$
28	WP	WP:WP3680	Physico-chemical features and toxicity-associated pathways	$2.095\times 10^{-4}$

## Data Availability

The genome assembly data are not available due to privacy reasons. The variants calling file and all needed data for performing the analysis and scripts used for it are available from https://github.com/shaileshp51/OUDgwas (accessed on 8 November 2024).

## Abbreviations

The following abbreviations are used in this manuscript:

DSM-IV	Diagnostic and Statistical Manual of Mental Disorders 4th edition
GWAS	Genome-wide association study
DSM	Diagnostic and Statistical Manual of Mental Disorders
OAD	Opioid addiction disorder
GOI	Genes of interest
SNP	Single nucleotide polymorphism
PPI	Protein-protein interaction
GO	Gene ontology

## References

[1] Florence C., Luo F., Rice K. (2021). The economic burden of opioid use disorder and fatal opioid overdose in the United States, 2017. Drug Alcohol Depend..

[2] Wall R., Rehm J., Fischer B., Brand’s B., Gliksman L., Stewart J., Medved W., Blake J. (2000). Social costs of untreated opioid dependence. J. Urban Health.

[3] Hedegaard H., Miniño A.M., Spencer M.R., Warner M. (2021). Drug overdose deaths in the United States, 1999–2020. NCHS Data Brief..

[4] Florence C.S., Zhou C., Luo F., Xu L. (2016). The Economic Burden of Prescription Opioid Overdose, Abuse, and Dependence in the United States, 2013. Med. Care.

[5] Cheung A., Marchand J., Mark P. (2022). Loss of Life and Labor Productivity: The Canadian Opioid Crisis. Ann. Am. Acad. Pol. Soc. Sci..

[6] Robertson A.G., Easter M.M., Lin H.J., Frisman L.K., Swanson J.W., Swartz M.S. (2018). Associations between pharmacotherapy for opioid dependence and clinical and criminal justice outcomes among adults with co-occurring serious mental illness. J. Subst. Abus. Treat..

[7] Grella C.E., Ostile E., Scott C.K., Dennis M., Carnavale J. (2020). A Scoping Review of Barriers and Facilitators to Implementation of Medications for Treatment of Opioid Use Disorder within the Criminal Justice System. Int. J. Drug Policy.

[8] Tsuang M.T., Lyons M.J., Meyer J.M., Doyle T., Eisen S.A., Goldberg J., True W., Lin N., Toomey R., Eaves L. (1998). Co-occurrence of abuse of different drugs in men: The role of drug-specific and shared vulnerabilities. Arch. Gen. Psychiatry.

[9] Kendler K.S., Karkowski L.M., Neale M.C., Prescott C.A. (2000). Illicit psychoactive substance use, heavy use, abuse, and dependence in a US population-based sample of male twins. Arch. Gen. Psychiatry.

[10] Tsuang M.T., Lyons M.J., Eisen S.A., Goldberg J., True W., Lin N., Meyer J.M., Toomey R., Faraone S.V., Eaves L. (1996). Genetic influences on DSM-III-R drug abuse and dependence: A study of 3,372 twin pairs. Am. J. Med. Genet..

[11] Haerian B.S., Haerian M.S. (2013). OPRM1 rs1799971 polymorphism and opioid dependence: Evidence from a meta-analysis. Pharmacogenomics.

[12] Beer B., Erb R., Pavlic M., Ulmer H., Giacomuzzi S. (2013). Association of Polymorphisms in Pharmacogenetic Candidate Genes (OPRD1, GAL, ABCB1, OPRM1) with Opioid Dependence in European Population: A Case-Control Study. PLoS ONE.

[13] Zhou H., Rentsch C.T., Cheng Z., Kember R.L., Nunez Y.Z., Sherva R.M., Tate J.P., Dao C., Xu K., Polimanti R. (2020). Association of OPRM1 Functional Coding Variant with Opioid Use Disorder: A Genome-Wide Association Study. JAMA Psychiatry.

[14] Dunn K.E., Huhn A.S., Finan P.H., Mange A., Bergeria C.L., Maher B.S., Rabinowitz J.A., Strain E.C., Antoine D. (2024). Polymorphisms in the A118G SNP of the OPRM1 gene produce different experiences of opioids: A human laboratory phenotype–genotype assessment. Addict. Biol..

[15] Le Foll B., Gallo A., Le Strat Y., Lu L., Gorwood P. (2009). Genetics of dopamine receptors and drug addiction: A comprehensive review. Behav. Pharmacol..

[16] Chen D., Liu F., Shang Q., Song X., Miao X., Wang Z. (2011). Association between polymorphisms of DRD2 and DRD4 and opioid dependence: Evidence from the current studies. Am. J. Med. Genet. Part B Neuropsychiatr. Genet..

[17] Clarke T.K., Weiss A.R., Ferarro T.N., Kampman K.M., Dackis C.A., Pettinati H.M., O’brien C.P., Oslin D.W., Lohoff F.W., Berrettini W.H. (2014). The dopamine receptor D2 (DRD2) SNP rs1076560 is associated with opioid addiction. Ann. Hum. Genet..

[18] Bawor M., Dennis B.B., Tan C., Pare G., Varenbut M., Daiter J., Plater C., Worster A., Marsh D.C., Steiner M. (2015). Contribution of BDNF and DRD2 genetic polymorphisms to continued opioid use in patients receiving methadone treatment for opioid use disorder: An observational study. Addict. Sci. Clin. Pract..

[19] Crist R., Ambrose-Lanci L., Vaswani M., Clarke T., Zeng A., Yuan C., Ferraro T., Hakonarson H., Kampman K., Dackis C. (2013). Case–control association analysis of polymorphisms in the delta-opioid receptor, OPRD1, with cocaine and opioid addicted populations. Drug Alcohol Depend..

[20] Lutz P.E., Kieffer B.L. (2013). The multiple facets of opioid receptor function: Implications for addiction. Curr. Opin. Neurobiol..

[21] Miranda M., Morici J.F., Zanoni M.B., Bekinschtein P. (2019). Brain-derived neurotrophic factor: A key molecule for memory in the healthy and the pathological brain. Front. Cell. Neurosci..

[22] Liu Q.R., Walther D., Drgon T., Polesskaya O., Lesnick T.G., Strain K.J., De Andrade M., Bower J.H., Maraganore D.M., Uhl G.R. (2005). Human brain derived neurotrophic factor (BDNF) genes, splicing patterns, and assessments of associations with substance abuse and Parkinson’s Disease. Am. J. Med. Genet. Part B Neuropsychiatr. Genet..

[23] Koo J.W., Mazei-Robison M.S., Chaudhury D., Juarez B., LaPlant Q., Ferguson D., Feng J., Sun H., Scobie K.N., Damez-Werno D. (2012). BDNF is a negative modulator of morphine action. Science.

[24] Nielsen D., Ji F., Yuferov V., Ho A., Chen A., Levran O., Ott J., Kreek M. (2008). Genotype patterns that contribute to increased risk for or protection from developing heroin addiction. Mol. Psychiatry.

[25] Nielsen D.A., Ji F., Yuferov V., Ho A., He C., Ott J., Kreek M.J. (2010). Genome-wide association study identifies genes that may contribute to risk for developing heroin addiction. Psychiatr. Genet..

[26] Gelernter J., Kranzler H.R., Sherva R., Koesterer R., Almasy L., Zhao H., Farrer L.A. (2014). Genome-wide association study of opioid dependence: Multiple associations mapped to calcium and potassium pathways. Biol. Psychiatry.

[27] Cheng Z., Zhou H., Sherva R., Farrer L.A., Kranzler H.R., Gelernter J. (2018). Genome-wide Association Study Identifies a Regulatory Variant of RGMA Associated With Opioid Dependence in European Americans. Biol. Psychiatry.

[28] Nelson E.C., Agrawal A., Heath A.C., Bogdan R., Sherva R., Zhang B., Al-Hasani R., Bruchas M.R., Chou Y.L., Demers C.H. (2016). Evidence of CNIH3 involvement in opioid dependence. Mol. Psychiatry.

[29] Crist R.C., Reiner B.C., Berrettini W.H. (2019). A review of opioid addiction genetics. Curr. Opin. Psychol..

[30] Gaddis N., Mathur R., Marks J., Zhou L., Quach B., Waldrop A., Levran O., Agrawal A., Randesi M., Adelson M. (2022). Multi-trait genome-wide association study of opioid addiction: OPRM1 and beyond. Sci. Rep..

[31] Gondré-Lewis M.C., Elman I., Alim T., Chapman E., Settles-Reaves B., Galvao C., Gold M.S., Baron D., Kazmi S., Gardner E. (2022). Frequency of the Dopamine Receptor D3 (rs6280) vs. Opioid Receptor µ1 (rs1799971) Polymorphic Risk Alleles in Patients with Opioid Use Disorder: A Preponderance of Dopaminergic Mechanisms?. Biomedicines.

[32] Freiermuth C.E., Kisor D.F., Lambert J., Braun R., Frey J.A., Bachmann D.J., Bischof J.J., Lyons M.S., Pantalon M.V., Punches B.E. (2023). Genetic variants associated with opioid use disorder. Clin. Pharmacol. Ther..

[33] Ho A.M., Tang N.L., Cheung B.K., Stadlin A. (2008). Dopamine receptor D4 gene -521C/T polymorphism is associated with opioid dependence through cold-pain responses. Ann. N. Y. Acad. Sci..

[34] Chung P., Logge W.B., Riordan B.C., Haber P.S., Merriman M.E., Phipps-Green A., Topless R.K., Merriman T.R., Conner T., Morley K.C. (2020). Genetic polymorphisms on OPRM1, DRD2, DRD4, and COMT in young adults: Lack of association with alcohol consumption. Front. Psychiatry.

[35] Pedrosa E., Kaushik S., Lachman H.M. (2010). ChIP-chip analysis of neurexins and other candidate genes for addiction and neuropsychiatric disorders. J. Neurogenet..

[36] Stoltenberg S.F., Lehmann M.K., Christ C.C., Hersrud S.L., Davies G.E. (2011). Associations among types of impulsivity, substance use problems and neurexin-3 polymorphisms. Drug Alcohol Depend..

[37] McLaren W., Gil L., Hunt S.E., Riat H.S., Ritchie G.R., Thormann A., Flicek P., Cunningham F. (2016). The ensembl variant effect predictor. Genome Biol..

[38] Purcell S., Neale B., Todd-Brown K., Thomas L., Ferreira M.A., Bender D., Maller J., Sklar P., De Bakker P.I., Daly M.J. (2007). PLINK: A tool set for whole-genome association and population-based linkage analyses. Am. J. Hum. Genet..

[39] Lie E., Ko J.S., Choi S.Y., Roh J.D., Cho Y.S., Noh R., Kim D., Li Y., Kang H., Choi T.Y. (2016). SALM4 suppresses excitatory synapse development by cis-inhibiting trans-synaptic SALM3–LAR adhesion. Nat. Commun..

[40] Lie E., Yeo Y., Lee E.J., Shin W., Kim K., Han K.A., Yang E., Choi T.Y., Bae M., Lee S. (2021). SALM4 negatively regulates NMDA receptor function and fear memory consolidation. Commun. Biol..

[41] Fill M., Copello J.A. (2002). Ryanodine receptor calcium release channels. Physiol. Rev..

[42] Santulli G., Nakashima R., Yuan Q., Marks A.R. (2017). Intracellular calcium release channels: An update. J. Physiol..

[43] Brini M., Calì T., Ottolini D., Carafoli E. (2014). Neuronal calcium signaling: Function and dysfunction. Cell. Mol. Life Sci..

[44] Li X., Zhu C., Tu W.H., Yang N., Qin H., Sun Z. (2011). ZMIZ1 preferably enhances the transcriptional activity of androgen receptor with short polyglutamine tract. PLoS ONE.

[45] Tobiansky D.J., Wallin-Miller K.G., Floresco S.B., Wood R.I., Soma K.K. (2018). Androgen regulation of the mesocorticolimbic system and executive function. Front. Endocrinol..

[46] Szklarczyk D., Kirsch R., Koutrouli M., Nastou K., Mehryary F., Hachilif R., Gable A.L., Fang T., Doncheva N.T., Pyysalo S. (2023). The STRING database in 2023: Protein–protein association networks and functional enrichment analyses for any sequenced genome of interest. Nucleic Acids Res..

[47] Raudvere U., Kolberg L., Kuzmin I., Arak T., Adler P., Peterson H., Vilo J. (2019). g: Profiler: A web server for functional enrichment analysis and conversions of gene lists (2019 update). Nucleic Acids Res..

[48] Bogari N.M., Al-Allaf F.A., Aljohani A., Taher M.M., Qutub N.A., Alhelfawi S., Alobaidi A., Alqudah D.M., Banni H., Dairi G. (2020). The co-existence of ADHD with autism in Saudi children: An analysis using next-generation DNA sequencing. Front. Genet..

[49] Van der Auwera G.A., O’Connor B.D. (2020). Genomics in the cloud: Using Docker, GATK, and WDL in Terra.

[50] Poplin R., Ruano-Rubio V., DePristo M.A., Fennell T.J., Carneiro M.O., Van der Auwera G.A., Kling D.E., Gauthier L.D., Levy-Moonshine A., Roazen D. (2017). Scaling accurate genetic variant discovery to tens of thousands of samples. bioRxiv.

[51] Caetano-Anolles D. (2023). (How to) Filter Variants Either with VQSR or by Hard-Filtering.

[52] Danecek P., Bonfield J.K., Liddle J., Marshall J., Ohan V., Pollard M.O., Whitwham A., Keane T., McCarthy S.A., Davies R.M. (2021). Twelve years of SAMtools and BCFtools. GigaScience.

[53] Shannon P., Markiel A., Ozier O., Baliga N.S., Wang J.T., Ramage D., Amin N., Schwikowski B., Ideker T. (2003). Cytoscape: A software environment for integrated models of biomolecular interaction networks. Genome Res..

